# Identification of *Soat1* as a Quantitative Trait Locus Gene on Mouse Chromosome 1 Contributing to Hyperlipidemia

**DOI:** 10.1371/journal.pone.0025344

**Published:** 2011-10-14

**Authors:** Zongji Lu, Zuobiao Yuan, Toru Miyoshi, Qian Wang, Zhiguang Su, Catherine C. Chang, Weibin Shi

**Affiliations:** 1 Department of Radiology and Medical Imaging, University of Virginia, Charlottesville, Virginia, United States of America; 2 Department of Biochemistry and Molecular Genetics, University of Virginia, Charlottesville, Virginia, United States of America; 3 Department of Biochemistry, Dartmouth Medical School, Hanover, New Hampshire, United States of America; Universitätsklinikum Schleswig-Holstein - Campus Luebeck, Germany

## Abstract

We previously identified two closely linked quantitative trait loci (QTL) on distal chromosome 1 contributing to major variations in plasma cholesterol and triglyceride levels in an intercross derived from C57BL/6 (B6) and C3H/HeJ (C3H) apolipoprotein E-deficient (apoE^−/−^) mice. *Soat1*, encoding sterol o-acyltransferase 1, is a functional candidate gene located underneath the proximal linkage peak. We sequenced the coding region of *Soat1* and identified four single nucleotide polymorphisms (SNPs) between B6 and C3H mice. Two of the SNPs resulted in amino-acid substitutions (Ile147Val and His205Tyr). Functional assay revealed an increased enzyme activity of *Soat1* in peritoneal macrophages of C3H mice relative to those of B6 mice despite comparable protein expression levels. Allelic variants of *Soat1* were associated with variations in plasma cholesterol and triglyceride levels in an intercross between B6.apoE^−/−^ and C3H.apoE^−/−^ mice. Inheritance of the C3H allele resulted in significantly higher plasma lipid levels than inheritance of the B6 allele. *Soat1* variants were also significantly linked to major variations in plasma esterified cholesterol levels but not with free cholesterol levels. Trangenic expression of C3H *Soat1* in B6.apoE^−/−^ mice resulted in elevations of plasma cholesterol and triglyceride levels. These results indicate that *Soat1* is a QTL gene contributing to hyperlipidemia.

## Introduction

Hyperlipidemia, comprising elevated levels of plasma cholesterol, triglyceride, or both, is a major risk factor for atherosclerotic cardiovascular disease [1]. Although a small subset of hyperlipidemia cases are caused by rare mutants that result in Mendelian traits segregating in families, the common forms of hyperlipidemia involve multiple genes and exhibit significant gene-environment interactions [2]. Recent genome-wide association studies (GWAS) have been remarkably successful in identifying novel genetic loci contributing to lipid metabolism [3], although it is challenging to establish causality between a genetic variant and trait in humans due to small gene effect, complex genetic structure, and environmental influences.

One effective approach to the identification of complex trait genes is the use of mouse models. Apolipoprotein E-deficient (apoE^−/−^) mice develop spontaneous hyperlipidemia and atherosclerosis even when fed a low-fat diet [4],[5]. Using intercrosses derived from apoE^−/−^ mouse strains, we and others have identified distal chromosome 1 as a major region contributing to hyperlipidemia [6],[7],[8]. QTL analysis of an intercross derived from C57BL/6 (B6) and C3H/HeJ (C3H) apoE^−/−^ mice has suggested that two closely linked loci in the distal chromosome 1 region account for major variations in plasma HDL, non-HDL cholesterol, and triglyceride levels [7]. The distal locus corresponds to *Hdlq5*, a HDL QTL identified in advanced intercross lines derived from B6 and NZB/B mice [9]. Subsequent studies have identified *Apoa2* as the causative gene of *Hdlq5* [10]. The proximal locus overlaps with *Cq1* (158.6 Mbp), a locus identified in a B6×KK-Ay intercross for plasma cholesterol concentrations [11]. *Soat1* is a functional candidate gene close to the linkage peak of the proximal QTL. It encodes an enzyme in the endoplasmic reticulum that catalyzes the formation of cholesteryl esters from cholesterol and fatty acyl coenzyme A [12]. In mammals, two *Soat* genes have been identified: *Soat1* is ubiquitously expressed and is responsible for cholesteryl ester formation in the brain, adrenal glands, macrophages, kidneys, and the liver, and *Soat2* is expressed in the liver and intestines. *Soat1* deficiency results in a significant reduction in non-HDL levels of apoE^−/−^ and LDLR^−/−^ mice fed a chow or Western diet [13]. Human genetic studies indicate that *Soat1* variants are associated with elevations in plasma concentrations of HDL cholesterol and apoA-I among subjects with hyperlipidemia [14]. In the present study, we tested whether *Soat1* was a QTL gene contributing to naturally occurring variation in plasma lipid levels, especially under the circumstances of hyperlipidemia, in mice.

## Results

### Soat1 sequence variation

**Figure 1:**
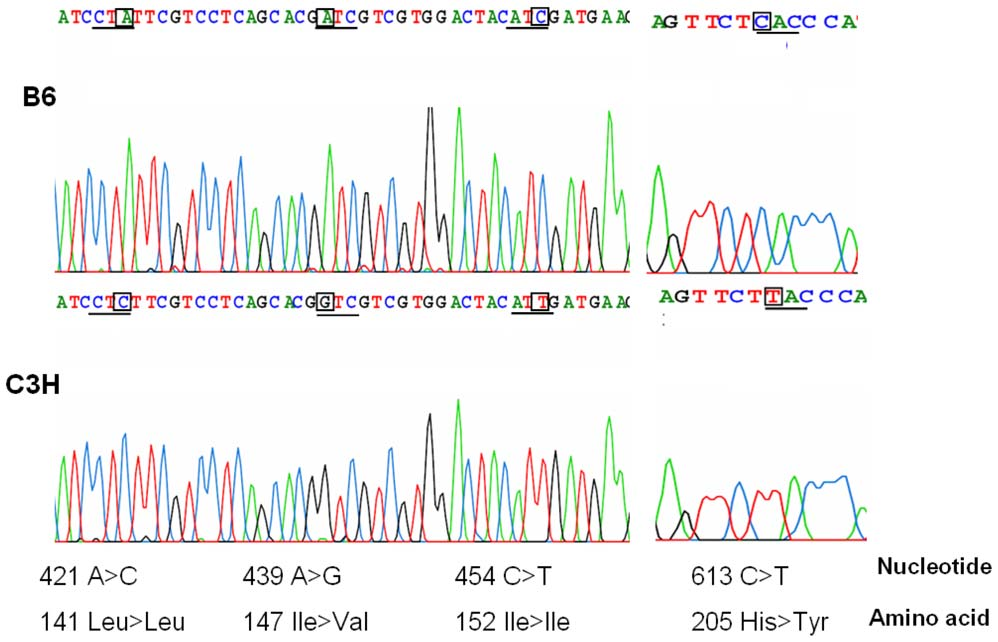
Selected sequence traces of *Soat1* cDNA of B6 and C3H mice. Differences between the two strains in nucleotides or amino acid residues are highlighted. Partial sequences of *Soat1* cDNA are not presented because no sequence difference has been found between the two strains.

**Figure 2:**
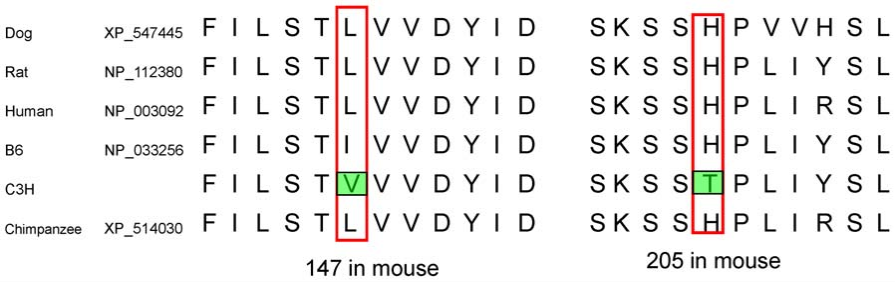
Comparison of *Soat1* protein sequences in five mammal species. Amino acid residues that are different between B6 and C3H mice are highlighted. GenBank accession No. are shown following the names of the species.

As part of an effort to find causal genes for the proximal lipid QTL on distal chromosome 1, all genes within the confidence interval (154.9∼172.8 Mb) were perused for sequence differences in coding or promoter regions between B6 and C3H mice by querying public accessible databases (http://www.ncbi.nlm.nih.gov/SNP/MouseSNP.cgi, http://cgd.jax.org/tools/diversityarray.shtml, and www.ensembl.org). 16 likely candidate genes whose sequence variations may lead to changes in either the structure or quantity of a gene product were identified, which included *Qsox1*, *Cep350*, *Tor1aip1*, *Tdrd5*, *Nphs2*, *Soat1*, *Tor3a*, *sec16b*, *Lztr2*, *Tnr*, *AI848100*, *LOC100040571*, *1700015E13Rik*, *Nos1ap*, *Olfml2b*, and *Atf6*. Among them, *Soat1* is a gene located close to the linkage peak and is also involved in lipid metabolism. We sequenced the coding region of *Soat1* by using cDNA as template and found four SNPs between B6 and C3H mice (Figure 1) (the accession number of the C3H/HeJ *Soat1* gene sequence in the NCBI GenBank is bankit838062 DQ903181). Two SNPs, A/G at position 439 and C/T at 613, led to amino-acid substitutions with isoleucine (Ile) to valine (Val) at amino acid residue 147 (Ile147Val) and histidine (His) to tyrosine (Tyr) at residue 205 (His205Tyr), respectively. The other two SNPs, A/C at 421 and C/T at 454, were synonymous base changes. We compared *Soat1* peptide sequences of five mammal species, including the mouse, and found that 205His is conserved in humans, chimpanzees, dogs, rats, and B6 mice (Figure 2), suggesting that 205Tyr is a mutation in C3H mice. The nonsynonymous SNP at 439 also represents a conservative change from leucine to isoleucine in B6 or to valine in C3H.

### Enhanced enzyme activity

**Figure 3:**
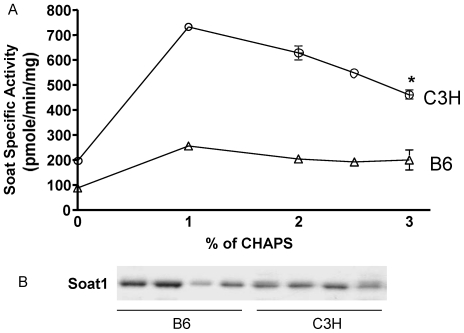
Comparison of *Soat1* enzyme activity in peritoneal macrophages harvested from B6.apoE^−/−^ and C3H.apoE^−/−^ mice (A). Cell homogenates were prepared from five individual mice of each strain and kept at concentrations of 2–4 mg/ml in a buffer (50 mM Tris, 1 mM EDTA at pH 7.8 with protease inhibitors). To solubilize the enzyme, 1 M KCl and various concentrations of CHAPS were added. The enzyme was assayed in duplicate in taurochoate/cholesterol/PC mixed micelles. *P<0.05. C3H had significantly higher *Soat1* activity levels than B6. Western blot analysis of *Soat1* expression in peritoneal macrophages derived from B6.apoE^−/−^ and C3H.apoE^−/−^ mice (B). Each lane represents an individual mouse. There was no significant difference in *Soat1* expression between the two strains.

*Soat1* activity was determined *in vitro* using cell homogenates prepared from peritoneal macrophages of B6.apoE^−/−^ and C3H.apoE^−/−^ mice. The enzyme activity was optimally solubilized with the zwitterionic detergent CHAPS at concentrations of 1–3% (Figure 3A). At all the concentrations used, the Soat specificity activity was nearly twice as high in C3H as in B6 (*P*<0.05). The expression of *Soat1* in peritoneal macrophages was examined by western blot analysis (Figure 3B). Densitometry of *Soat1* bands was comparable between the two strains [442±60 vs. 421±115 (optical density); *P* = 0.87].

### Association with variation in plasma lipid levels

We then examined whether *Soat1* variants were associated with variations in plasma HDL, non-HDL cholesterol, and triglyceride levels in female F_2_ mice derived from B6.apoE^−/−^ and C3H.apoE^−/−^ mice. As shown in Table 1, inheritance of two copies of the C3H allele (CC genotype) resulted in significantly higher triglyceride, HDL, and non-HDL cholesterol levels than inheritance of two copies of the B6 allele (BB genotype) at the *Soat1* locus (*P*<0.05 for each trait).

**Table 1:** Statistical association between allelic variation at the *Soat1* locus and plasma lipid levels in F_2_ mice derived from B6.apoE^−/−^ and C3H.apoE^−/−^ mice.

Trait	BB (n = 61)	BC (n = 112)	CC (n = 45)	Variance (%)	P value
Triglyceride	154±37	175±44	185±52	5	2.1×10^−3^
Non-HDL	701±187	803±201	911±262	10	1.0×10^−5^
HDL	26±20	38±30	38±27	3	3.5×10^−2^

Measurements are presented as means ± SD. The unit for these measurements is mg/dl. Variance (%) accounted for by the *Soat1* locus is expressed as the percentage of the total phenotypic variance detected in the F_2_ cohort. BB, homozygous for C57BL/6 alleles; CC, homozygous for C3H alleles; BC, heterozygous for C57BL/6 and C3H alleles. The percentage of variance explained by *Soat1* genotype and likelihood ratio P values are shown.

### Linkage to plasma esterified cholesterol

**Figure 4:**
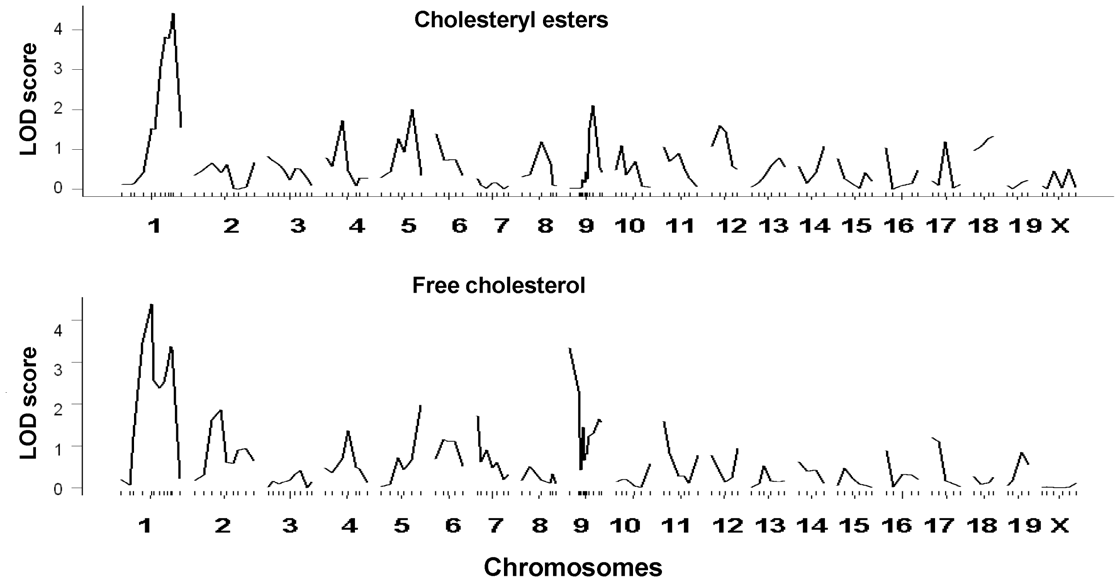
Genome-wide analyses for main effect loci affecting plasma esterified cholesterol and free cholesterol of female F_2_ mice between B6.apoE^−/−^ and C3H.apoE^−/−^ mice using the R/qtl program. Female F_2_ mice were fed the western diet for 12 weeks and typed for genetic markers spanning the genome and for the phenotypes. Chromosomes 1 through X are represented numerically on the X-axis. The relative width of the space allotted for each chromosome reflects the relative length of each chromosome. The Y-axis represents the LOD score.

**Figure 5:**
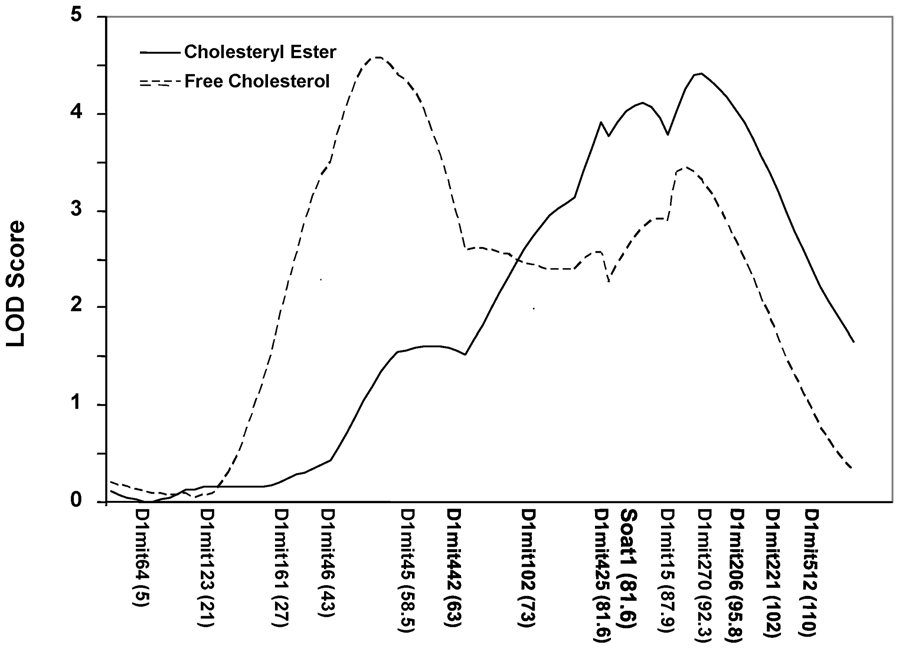
LOD score plots for esterified and free cholesterol levels on chromosome 1. The x-axis depicts the marker positions in centimorgans, and the y-axis depicts the LOD score. The microsatellite markers typed are listed below the x-axis, corresponding to their map locations on the chromosome.

Because *Soat1* is an enzyme that catalyzes free cholesterol to cholesterol esters, we determined whether the *Soat1* locus was linked to variations in plasma esterified and free cholesterol levels in the BXH cross. QTL analysis of F_2_ mice revealed that loci on chromosome 1 were responsible for major variations in plasma esterified cholesterol and free cholesterol levels (Figure 4). The interval mapping graph for chromosome 1 showed that the proximal peak of linkage curves for esterified cholesterol overlapped precisely with the *Soat1* locus (Figure 5), which had a LOD score of 4.2 and explained 8% of the variance (Table 2). The distal peak of linkage curves appeared near marker *D1Mit206* (174.9 Mbp), which had a LOD score of 4.4 and accounted for 9% of the variance. In contrast, free cholesterol was controlled by two significant QTLs on chromosome 1, near markers *D1Mit45* (94.9 Mbp) and *D1Mit270* (172.7 Mbp), respectively, and a suggestive locus near marker *D9Mit297* (33.8 Mbp) on chromosome 9. The QTL near marker *D1Mit45* had a significant LOD score of 4.6 and accounted for 9% of the variance, and the QTL near marker *D1Mit270* had a LOD score of 3.4 and explained 7% of the variance. The suggestive QTL near marker *D9Mit297* (33.9 Mbp) for free cholesterol had a LOD score of 3.3 and accounted for 6% of the variance, and this QTL overlaps with *Cq4* and *Cq5* mapped in B6×KK-A F2 and KK×RR F2 crosses [11].

**Table 2:** Significant and suggestive QTL for plasma esterified and free cholesterol in F_2_ mice derived from B6.apoE^−/−^ and C3H.apoE^−/−^ mice.

*Chromosome marker (cM)* a	*Trait*	*LOD* b	*SI (cM)* c	*Variance (%)* d	P_emp_ e	*Model of inheritance* f
Soat1 (81.6)	Esterified cholesterol	4.2	75–89	8	0.002	Additive
D1mit206(95.8)	Esterified cholesterol	4.4	87–102	9	0.001	Additive
D1mit45(58.5)	Free cholesterol	4.6	41–62	9	0.004	Additive
D1mit270(92.3)	Free cholesterol	3.4	73–101	7	0.005	Additive
D9mit297(15)	Free cholesterol	3.3	0–27	6	0.008	Additive

a From Mouse Genome Informatics database at http://www.informatics.jax.org.

b Suggestive QTL and significant QTL were 2.4 and 3.4, respectively, for free cholesterol and 2.4 and 3.3, respectively, for esterified cholesterol as defined by 1000 permutation tests.

c Support intervals (SI) were defined by a 1-unit decrease in LOD score on either side of the peak marker.

d Variance (%) indicates the percentage of the phenotypic variance at the peak marker.

e *P*_emp_, empirically determined *P*-value for the whole genome, was calculated using the permutation test function of MapManager QTXb20.

f Model of inheritance was determined using the MapManager QT program. C3H was the high allele at all the markers, contributing to elevated free or esterified cholesterol levels.

### Analysis of transgenic mice

**Figure 6:**
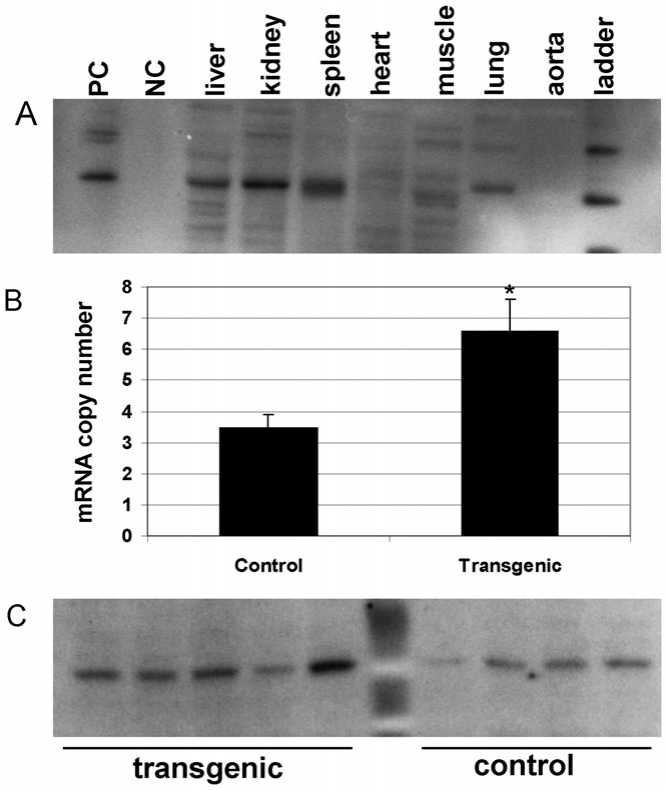
Characterization of *Soat1* transgenic mice. A, expression of *Soat1* protein in transgenic mice. PC, positive control: lane loaded with *Soat1* protein; NC, negative control: lane loaded with buffer only. B, real-time PCR analysis of *Soat1* mRNA expression in the liver of transgenic and non-transgenic (control) littermates. The expression level of *Soat1* was expressed as copy number relative to 10,000 copies of GAPDH mRNA. Results are means ± SE of 6 and 5 transgenic and non-transgenic mice, respectively. C, expression of *Soat1* protein in the liver of transgenic and non-transgenic littermates. Each lane represents an individual mouse.

**Figure 7:**
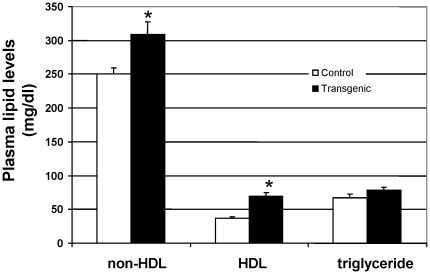
Plasma lipid levels of female transgenic and non-transgenic (control) littermates on the B6.apoE^−/−^ background. Blood was collected from overnight fasted mice fed a chow diet. Values are mean ± SE of 5 transgenic and 8 non-transgenic mice. * *P*<0.05 vs. non-transgenic littermates.

To directly evaluate the role of *Soat1* in hyperlipidemia, we constructed transgenic mice that expressed C3H *Soat1* and crossed the mice with B6.apoE^−/−^ mice for more than six generations. The expression of *Soat1* protein in transgenic mice was analyzed by western blotting, and it was found in the liver, kidney, spleen, and the lung but not in the aorta, heart, or skeletal muscle (Figure 6). Real-time PCR analysis revealed that *Soat1* mRNA expression levels in the liver were 2-fold as high in transgenic mice as in non-transgenic littermates (6.57±1.04 vs. 3.47±0.44 per 10,000 copies of GAPDH; *P* = 0.029). The mRNA expression level of GAPDH was comparable between transgenic and non-transgenic mice with respect to real-time PCR cycle threshold values (18.17±0.32 vs. 18.31±0.28). *Soat1* protein in the liver was higher in transgenic mice than non-transgenic littermates (optical density: 6175262±1338918 vs. 4270654±1017286), although the difference was not statistically significant (*P* = 0.295). Compared to non-transgenic littermates, transgenic mice had significantly elevated plasma levels of non-HDL cholesterol (308.6±18.5 vs. 250.6±8.4 mg/dl; *P* = 0.029) and HDL cholesterol (68.9±6.1 vs. 37.2±2.3 mg/dl; *P* = 0.0048) (Figure 7). Plasma triglyceride levels were also higher in transgenic mice (78.1±5.1 vs. 67.0±5.5 mg/dl), although the difference was not statistically significant (*P* = 0.17).

## Discussion

QTLs for plasma HDL on mouse distal chromosome 1 have been reported many times in numerous crosses [15]. QTLs for plasma triglyceride and non-HDL on distal chromosome 1 have also been reported in several crosses, including three BXH crosses [6],[7],[8],[16]. In the intercross between B6.apoE^−/−^ and C3H.apoE^−/−^ mice, we have observed that QTLs for HDL coincide with QTLs for triglyceride and non-HDL on distal chromosome 1 [7], suggesting that these plasma lipid phenotypes are controlled by the same genes. We have also observed two distinct peaks of the linkage curves for plasma triglyceride or non-HDL with the distal peak near marker *D1Mit270* (172.7 Mbp) and the proximal peak near marker *D1Mit425* (158.6 Mbp) [7], suggesting the existence of two QTLs for plasma lipids in the distal chromosome 1 region. Two other studies have also suggested the existence of a lipid QTL near 81.6 cM on mouse chromosome 1 [8], [11].

The distal QTL overlaps with *Hdlq5* [9], and there is conclusive evidence supporting *Apoa2* (92.6 cM) to be the causal gene for the QTL. Indeed, sequence analysis of the *Apoa2* coding region in many mouse strains has revealed a number of nucleotide differences [10], [17]. *Apoa2* variants are associated with variation in HDL cholesterol levels of mice [10],[18]. Transgenic overexpression of *Apoa2* elevates plasma HDL, non-HDL cholesterol, and triglyceride levels [19], and *Apoa2* deficiency reduces plasma HDL, non-HDL, and triglyceride levels in mice [20].

The present study strongly suggests that *Soat1* is the causal gene for the proximal QTL. Multiple polymorphisms have been found in the coding region of this gene between the two parental strains that had been used in our previous studies to map the lipid QTL, and two of the polymorphisms lead to amino acid substitutions in the protein product. These polymorphisms led to changes in the function of *Soat1* enzyme. As macrophages express only *Soat1*, we measured its activity in these cells. The present finding that C3H had significantly higher Soat enzyme activity than B6 despite comparable protein expression in macrophages indicates that these SNPs have resulted in increases in Soat activity. This study has also provided several lines of other evidence supporting *Soat1* to be a QTL gene affecting plasma lipid levels. First, the parental strain C3H, which has higher *Soat1* enzyme activity, exhibits higher plasma cholesterol and triglyceride levels than the B6 strain, which has lower enzyme activity [21]. Second, F_2_ mice with the C3H allele at the *Soat1* locus had higher plasma cholesterol and triglyceride levels than those with the B6 allele. Third, the QTL for esterified cholesterol coincided with the QTLs for triglyceride, HDL, and non-HDL at the *Soat1* locus, suggesting that a gene involved in cholesterol esterification affected plasma HDL and non-HDL cholesterol levels. *Soat1* is such a gene that synthesizes cholesterol esters and potentially affects lipoprotein assembling. Finally, the direct evidence is that transgenic mice expressing C3H *Soat1* had significantly elevated plasma levels of HDL and non-HDL cholesterol.

A previous study also showed that *Soat1* deficiency reduces plasma total cholesterol levels of apoE^−/−^ mice [13]. However, the previous knockout mice were generated using embryonic stem cells derived from 129/SvJ mice and then backcrossed onto the B6 background. Linkage would cause the retention of a significant segment of 129/SvJ chromosome harboring the targeted gene in recipient mice. Several genes adjacent to *Soat1*, including *Apoa2*, are polymorphic between B6 and 129/SvJ and could contribute to variations in plasma lipid levels. The present study of transgenic mice has excluded a possible interference from *Apoa2* and thus provided more definite evidence on the role of *Soat1* in modifying plasma lipid levels. In this study, we found that transgenic mice had an increased level of HDL cholesterol. In contrast, the effect of *Soat1* on plasma HDL cholesterol was not observed in the knockout mice. An explanation for the discrepant results is that apoE^−/−^ mice have an extremely low HDL cholesterol level, and thus it would be harder for a gene to exert an effect to further reduce than to elevate it. In humans, a missense variant (R526G) and a variant in the 5′ untranslated region (−77G>A) have been found in the *Soat1* gene, and individuals with −77G>A variant have significantly higher plasma HDL concentrations than those without the variant among hyperlipidemic subjects [14]. Nevertheless, genome-wide association studies (GWAS) to date have failed to detect any association with lipid traits in humans. One probable explanation for this outcome is that the gene is large (64.89 kb) and includes many variants (794 SNPs in the NCBI dbSNP database). Only a small number of markers in Soat1 have been typed, and these markers may not have the strongest association.

In summary, we have provided reasonable evidence to support *Soat1* to be a QTL gene, although further studies are needed to prove which SNP affects Soat1 function. Soat enzyme catalyzes the formation of cholesteryl esters from free cholesterol and fatty acyl coenzyme A. Because of its potential role in foam cell formation, this enzyme has been a target for developing therapeutic drugs for the past two decades. Theoretically, inhibition of the enzyme should block the esterification of cholesterol and prevents the transformation of macrophages into foam cells. However, some animal studies show that Soat inhibitors exert hypolipidemic effects and reduce atherosclerosis [22],[23],[24] while some studies show that inhibition of Soat activity promotes atherosclerosis [25]. In humans, administration of Soat inhibitors failed to reduce, rather increase, plaque volume [26],[27]. Studies with more specific inhibitors of *Soat1* activity have also shown aggravation rather than alleviation of atherosclerosis in rabbits and mice [25],[28]. As *Soat1* elevates plasma levels of both good (HDL) and bad (non-HDL) cholesterol, the present results may explain why *Soat1* inhibitors are not clinically effective as expected and many have failed in clinical stages probably due to its effect on HDL cholesterol. Moreover, continued inhibition of Soat activity increases intracellular free cholesterol and limits the efflux of free cholesterol, which may induce cytotoxic effects within cells.

## Materials and Methods

### Ethics statement

All procedures were carried out in accordance with current National Institutes of Health guidelines and approved by the University of Virginia Animal Care and Use Committee (Assurance #A3245-01, Animal Protocol #3109).

### Mice

B6.apoE^−/−^ mice were purchased from the Jackson Laboratory, and C3H.apoE^−/−^ mice were generated in our laboratory. The generation of F_2_ mice from B6.apoE^−/−^ and C3H.apoE^−/−^ mice was reported previously [7]. At 6 weeks of age, female F_2_ mice were started on a Western-type diet containing 21% butterfat, 34% sucrose, and 0.2% cholesterol (TD 88137, Harlan Laboratories) and maintained on the diet for 12 weeks. To generate transgenic mice, a clone containing the *Soat1* gene in the pTARBAC2.1 vector was picked from the CHORI-34 Mouse C3H/HeJ BAC library constructed by the Pieter De Jong's Laboratory at Children's Hospital Oakland Research Institute. This clone contained the entire *Soat1* gene, 142,565 bp upstream and 14,893 bp downstream from the 5′ and 3′ ends, respectively. The integrity of *Soat1* was confirmed through partial sequencing and restriction enzyme digestion before the purified BAC DNA was microinjected into B6D2 F_1_ fertilized eggs at a concentration of 1 *μ*g/ml. Transgenic founders were identified by PCR amplifications of both forward and reverse fragments of the BAC DNA with primers 5′-TCTTTCTCCGCACCCGACATAGAT-3′/5′-TTAGGAGCCACTGTGGTTAGCTGT-3′ and 5′-AAGTCAGAACTGTGGCTTGT-3′/5′-CAGCACTGGTTTAAT GTCC-3′. Positive transgenic mice were backcrossed with B6.apoE^−/−^ mice for more than 6 generations and maintained in a heterozygous condition for the transgene.

### Plasma lipid measurements

Plasma total cholesterol, HDL cholesterol, and triglyceride were measured as reported previously [29]. Non-HDL cholesterol was calculated by subtracting the HDL cholesterol levels from the total. Free cholesterol levels were determined using a kit from Wako (Richmond, VA). Briefly, 6 µl of plasma samples, lipid standards, and controls were loaded in a 96-well plate and then mixed with 150 µl of free cholesterol reagent. After a 5-min incubation at 37°C, the absorbance at 600 nm was read on a Molecular Devices (Menlo Park, CA) plate reader. Esterified cholesterol levels were calculated by subtracting free cholesterol levels from total cholesterol levels.

### Soat1 cDNA sequencing and genotyping

Total RNA isolated from the liver of B6 mice and C3H mice was reverse transcribed to cDNA with use of the Superscript RT-PCR kit (Invitrogen). The PCR primers used for amplification of *Soat1* cDNA in both directions were as follows: 5′-AGGAAGCTTGATTGATAGTGG-3′/5′-CTTGGGTAGTTGTCTCGGTAA-3′; 5′-GCAAAGATCCACTACCCACAG-3′/5′-AACACGTACCGACAAGTCCAG-3′. After purification with a QIAquick PCR purification kit, PCR products were sequenced on an ABI Prism Cycle sequencer 310 (Applied Biosystems). The *Soat1* polymorphism at T454C was used to screen the BXH intercross with the PCR-restriction fragment length polymorphism (PCR-RFLP)-based method. PCR amplification on genomic DNA was performed using primers 5′-AGATTGTCCCTCTAAGGCGCCAA-3′ and 5′-AGCCTAGCTGGACCACTTTCTCAA-3′, and the 712 bp amplicon was then digested with *Cla*I restriction enzyme (New England BioLabs, Hertfordshire, UK) according to the manufacturer's instruction. The PCR product from the B6 allele but not that from the C3H allele was digested by *Cla*I to 511 bp and 201 bp fragments. Thus, the BB allele should exhibit two bands, the CC allele one band, and the BC alleles three bands on an agarose gel.

### Soat1 activity assay in macrophages

Peritoneal macrophages were prepared as we previously described [30]. Following a brief culture as a monolayer in RPMI medium containing 10% fetal bovine serum, macrophages were harvested by hypotonic shock and scraping, and the resultant cell homogenate was kept in a buffer (50 mM Tris, 1 mM EDTA at pH 7.8 with protease inhibitors) at concentrations of 2–4 mg/ml. To solubilize the enzyme, 1 M KCl and various concentrations of CHAPS were added. The enzyme activity of *Soat1* was measured in duplicate in taurochoate/cholesterol/PC mixed micelles as described by Chang et al [31].

### Western blot analysis

The presence of *Soat1* in various tissues of transgenic mice and in peritoneal macrophages of B6.apoE^−/−^ and C3H.apoE^−/−^ mice was determined by western blot analysis. Proteins were prepared as we previously described [32], separated by electrophoresis on 4–12% Tris-polyacrylamide gels, and electrophoretically transferred to nitrocellulose membranes. The membrane was probed with a rabbit polyclonal antibody against *Soat1* (H-125, Santa Cruz), and signals were detected by the chemiluminescence method (Invitrogen). The density of the bands was quantified with a densitometer (Molecular Devices, CA).

### Real-time PCR analysis of Soat1 expression

Total RNA extracted from the liver of transgenic mice was treated with DNase I, and then reverse transcribed to cDNA as described above. cDNA was mixed with SYBR Green supermix reagent (Bio-Rad) and specific primers to assess expression of *Soat1* and glyceraldehyde-3-phosphate dehydrogenase (GAPDH) by real-time PCR. Primers used for *Soat1* amplification were 5′-TGTGCATCAGAAAGGTACCACGGA-3′/5′-GTTGCCAGGAAACCACCAAAGTGA-3′ and for GAPDH were 5′-GGTGTGAACGGA TTTGGCCGTATT-3′/5′-GGCCTTGACTGTGCCGTTGAATTT-3′. Real-time PCR on each sample was run in triplicate on an iCycler iQ5 machine (Bio-Rad) under the condition of 50°C for 2 minutes, 95°C for 2 minutes, then 95°C 30 seconds, 60°C 30 seconds, and 72°C 30 seconds for 40 cycles as reported [33]. The expression level of *Soat1* was expressed as mRNA copy number relative to 10,000 copies of GAPDH mRNA.

### Statistical analysis

QTL analysis was performed as we previously described [7],[33] ANOVA was used for determining if the mean phenotype values of progeny with different genotypes at a specific marker were significantly different. The Student *t* test was used when only two means were compared. Differences were considered statistically significant at *P*<0.05.
